# Novel grading system for ischemia‒reperfusion injury manifestations in patients with acute ST-segment elevation myocardial infarction undergoing percutaneous coronary intervention

**DOI:** 10.1038/s41598-022-24019-6

**Published:** 2022-11-11

**Authors:** Xiaotong Wang, Binbin Li, Yue Hu, Shengjue Xiao, Minjia Guo, Tao Xu, Huimin Wu, Chunyan Huan, Jie Yin, Hong Zhu, Defeng Pan

**Affiliations:** 1 Department of Cardiology, The Affiliated Hospital of Xuzhou Medical University, 99 Huaihai West Road, Xuzhou, 221004 Jiangsu China; 2 Department of Cardiology, The Second Affiliated Hospital of Xuzhou Medical University, Xuzhou, 221006 Jiangsu China; 3 Department of General Medicine, The Affiliated Hospital of Xuzhou Medical University, Xuzhou, 221004 Jiangsu China; 4 Department of Cardiology, Zhongda Hospital, School of Medicine, Southeast University, Nanjing, 210009 Jiangsu China

**Keywords:** Cardiology, Signs and symptoms

## Abstract

To establish a simple myocardial ischemia‒reperfusion injury (MIRI) manifestation grading system based on clinical manifestations and coronary angiography during primary percutaneous coronary intervention (PPCI). All STEMI patients treated with PPCI from June 2018 to November 2019 were included. According to the MIRI manifestation grade, patients were divided into four grades (I–IV). Laboratory and clinical indicators of the patients and the occurrence of major adverse cardiac events (MACEs) within one year of follow-up were analyzed. A total of 300 patients were included. The higher the MIRI manifestation grade, the lower was the high-density lipoprotein cholesterol (HDL-C); the higher were the C-reactive protein (CRP), lipoprotein(a) [LP(a)], and peak levels of high-sensitivity troponin T (hs-cTnT), creatine kinase (CK-MB), and N-terminal pro-B-type natriuretic peptide (NT-proBNP); and the higher were the proportions of right coronary artery (RCA) and multivessel lesions (*P* < 0.05). The left ventricular end-diastolic dimension (LVEDD) and E/e′ values of patients with higher grades were significantly increased, while the LVEF, left ventricular short-axis functional shortening (LVFS) and E/A values were significantly decreased (*P* < 0.05). The one-year cumulative incidence of major adverse cardiac events (MACEs) in patients with grade I–IV disease was 7.7% vs. 26.9% vs. 48.4% vs. 93.3%, respectively, *P* < 0.05. The higher the MIRI manifestation grade, the more obvious is the impact on diastolic and systolic function and the higher is the cumulative incidence of MACEs within one year, especially in patients with multivessel disease, low HDL-C, high CRP, high LP(a) levels, and the RCA as the infarction-related artery.

## Introduction

ST segment elevation myocardial infarction (STEMI) is one of the clinical subtypes of AMI with the highest morbidity and mortality. Primary percutaneous coronary intervention (PPCI) is the most effective treatment for STEMI patients. However, the process of an opening an infarct-related artery (IRA) and restoring blood flow to the ischemic myocardium itself will also lead to further death of myocardial cells, that is, myocardial ischemia‒reperfusion injury (MIRI)^1^. Nearly 50% of the final myocardial infarct size is due to myocardial reperfusion injury (RI) and can therefore be reduced^2^; reperfusion injury seriously weakens the benefits of reperfusion.

Ischemia‒reperfusion injury is inevitable, but how to evaluate the degree of myocardial injury caused by reperfusion is still an unsolved problem in the clinic. The most effective method is to compare myocardial imaging before PCI with that after PCI, which can effectively evaluate the degree of reperfusion injury. However, because myocardial imaging must be performed before PCI, there is a risk of delayed reperfusion, which is not practical in clinical practice^3^. In addition, it is uncertain whether the myocardial injury shown on preoperative and postoperative myocardial magnetic resonance (MR) has been caused by reperfusion in a particular patient. Moreover, MR increases the economic burden on patients to a certain extent. Therefore, it is necessary to establish a simple method to evaluate the degree of reperfusion injury manifestations in patients with acute STEMI.

During primary PCI, when the guidewire passes through the occlusive IRA or balloon predilation, patients will have different clinical features and coronary angiography manifestations. Some patients have stable vital signs, some patients have decreased heart rate and blood pressure; moreover, even malignant arrhythmia, no-reflow, and cardiogenic shock can occur. The mechanism by which these phenomena occur is unclear. However, these phenomena show the extent of reperfusion injury to some extent, and it is not clear whether they are related to the prognosis of patients. Clinical manifestations during myocardial reperfusion, to some extent, reflect the viable myocardium. During myocardial reperfusion, more obvious clinical manifestations may be associated with a more viable myocardium, resulting in a different prognosis from the clinical manifestations. If the clinical manifestations of reperfusion injury are positively associated with a poorer prognosis, we can intervene in advance to prevent the occurrence of these symptoms. Conversely, if there are clinically specific patient features with a better prognosis, we may not need to prevent the emergence of these clinical features. According to the clinical characteristics and coronary angiography of STEMI patients during PPCI, this study established a simple grading system to preliminarily evaluate the degree of MIRI manifestation and further analyze the efficacy and prognosis of patients with different grades to provide targeted treatment to patients with STEMI and improve the prognosis.

## Materials and methods

### Ethical statement

The study was conducted in accordance with the relevant guidelines and regulations of the Declaration of Helsinki, and informed consent was obtained from all participants and/or their legal guardians. In addition, the study was approved by the Ethics Committee of the Affiliated Hospital of Xuzhou Medical University (XYFY2018-LK037).

### Participants

All acute STEMI patients treated with PPCI at the Affiliated Hospital of Xuzhou Medical University from June 2018 to November 2019 were included. The inclusion criteria were as follows: (1) STEMI diagnosed based on the Third Universal Definition of Myocardial Infarction^4^; and (2) at least 18 years of age. The patient or her or his guardian consented to PCI. Exclusion criteria: (1) Myocardial ischemia time > 12 h; (3) prior MI; spontaneous recanalization of infarct-related vessels and previous stent implantation history; (4) other organic heart diseases (cardiomyopathy, valvular heart disease, congenital heart disease, etc.); (5) severe noncardiovascular diseases (chronic obstructive pulmonary disease, infectious disease, connective tissue disease, malignant tumor);

### Study grade and classification

Judgment of myocardial ischemia‒reperfusion injury^4^. (1) The clinical features of reperfusion: hypotension (blood pressure lower than 90/60 mmHg or SBP and/or DBP decreased more than 30% after reperfusion), requiring drug treatment or an intra-aortic balloon (IABP). Reperfusion arrhythmias (including frequent ventricular premature beats, bradycardia, atrioventricular block, accelerated idioventricular rhythm, etc.) require drug treatment or device support (defibrillation, temporary pacemaker implantation). (2) Coronary angiography showed no reflow (TIMI flow ≤ 2 excluding mechanical obstruction factors such as coronary artery spasm, acute occlusion, thrombosis, severe dissection, or residual stenosis). Myocardial blush grade (MBG) changes included no myocardial reperfusion (MBG 0 or 1) and myocardial reperfusion (MBG 2 or 3).

#### The MIRI manifestation grade criteria were as follows

Grade I: The patient’s vital signs were stable, and the blood pressure and heart rate remained unchanged; Grade II: Transient hypotension (less than 90/60 mmHg or SBP and/or DBP decrease ≤ 30%) and/or reperfusion arrhythmia that was not treated; TIMI 3, MBG 3. Grade III: persistent hypotension (lower than 90/60 mmHg, or SBP and/or DBP decrease > 30% or need for drug treatment) and/or reperfusion arrhythmia and need for drug treatment; transient TIMI: 2, MBG: 2; Grade IV: cardiogenic shock or malignant arrhythmia; TIMI ≤ 2, MBG: 0 or 1. A total of 300 patients met the inclusion criteria, including 78 female patients and 222 male patients. According to the grading criteria of MIRI, there were 52 cases of grade I, 93 cases of grade II, 122 cases of grade III, and 33 cases of grade IV.

### Data collection

Patient data, including name, sex, age, onset time, past medical history, etc., were collected and recorded. Biochemical indices were detected in all patients after admission. The levels of hs-cTnT, CK-MB, and NT-proBNP were monitored immediately, 12 h, and 2–4 days after admission to obtain the peak values. The patients were measured by the same ultrasonic doctor within three days after PCI with a GE Vivid E90 type color Doppler ultrasound diagnostic instrument. The left ventricular end-diastolic dimension (LVEDD), left ventricular ejection fraction (LVEF), left ventricular short-axis functional shortening (LVFS), peak value of early diastolic blood flow (E peak), peak velocity of late diastolic blood flow (A peak), and early diastolic velocity (e′) were measured at the level of the mitral valve tip, and E/A and E/e′ were calculated, in which LVEF was measured by the Simpson method. The incidence of MACEs within one year after PCI was followed up. MACEs included angina, recurrent myocardial infarction, arrhythmia, heart failure, and all-cause death.

### Statistical analysis

SPSS 26.0 was used to analyze the data. The measurement data were tested by normality, and data that were in accordance with a normal distribution are all expressed as the mean ± standard deviation (m ± sd), indicating that univariate analysis of variance was used for comparisons between groups, and the Student–Newman–Keuls (SNK) method was used for pairwise comparisons. Data that did not conform to a normal distribution are expressed as the median (quartile). The Kruskal‒Wallis H test was used for comparisons between groups, and the Nemenyi method was used for pairwise comparisons. The number of cases (percentage) was used to express classification and count data, and comparison between groups was performed by analyzing the data by the χ^2^ test, pairwise comparison and the χ^2^ division method. Kaplan‒Meier survival analysis was used to compare the cumulative MACE incidence and mortality among different groups. The test levels were all set at *P* < 0.05 for statistical significance.

## Results

### Patient characteristics

A total of 300 patients were included. The number and proportion of MIRI manifestations or grade I to IV patients were 52 (17.3%), 93 (31%), 122 (40.7%), and 33 (11%), respectively. Age, sex, heart rate, history of hypertension, diabetes history, smoking history, and onset time were not statistically significant (*P* > 0.05). The level of HDL-C in patients with grade IV was significantly lower than that in patients with grades I, II, and III. The levels of CRP and LP(a) in patients with grade IV were significantly higher than those in patients with grade I and II (*P* < 0.05); there were no significant differences in LDL-C, TG, TC, Glu, and HbA1c levels among all grades of patients (*P* > 0.05). The proportion of LCX in patients with grade I was significantly higher than that in patients with grades II and IV. The proportion of RCA in patients with grades III and IV was significantly higher than that in patients with grades I and II. The proportion of multivessel lesions in patients with grade IV was significantly higher than that in patients with grade I (*P* < 0.05); there was no significant difference in the proportion of patients with single-vessel disease and double-vessel disease (*P* > 0.05) (Table 1).

**Table 1:** Baseline characteristics of the participants.

	I (*n* = 52)	II (*n* = 93)	III (*n* = 122)	IV (*n* = 33)	*F/χ*^*2*^	*P*
Sex (M/W)	39/13	76/17	84/38	23/10	4.906	0.179
Age	62.08 ± 10.92	60.17 ± 10.51	61.29 ± 14.02	63.79 ± 13.52	0.759	0.518
Ischemic time (h)	5 (3, 7)	4 (3, 7)	5 (3, 7)	5 (4, 6)	1.368	0.713
Hypertension	18 (34.6)	44 (47.3)	58 (47.5)	15 (45.5)	2.788	0.426
Diabetes	10 (19.2)	20 (21.5)	19 (15.6)	3 (9.1)	3.089	0.378
Smoking	20 (38.5)	36 (38.7)	53 (43.4)	10 (30.3)	2.003	0.572
HR, bpm	75.37 ± 11.86	76.17 ± 10.22	76.84 ± 13.93	75.82 ± 19.97	0.168	0.918
HDL-C (mmol/L)	1.04 ± 0.25	1.04 ± 0.25	1.1 ± 0.32	0.92 ± 0.18^abc^	3.521	0.015
LDL-C (mmol/L)	2.84 ± 0.92	2.87 ± 0.96	2.72 ± 0.86	2.9 ± 0.74	0.637	0.592
TC (mmol/L)	4.39 ± 1.04	4.45 ± 1.17	4.4 ± 1.02	4.34 ± 1	0.093	0.964
CRP (mg/L)	7.8 (4.3, 14.8)	8.1 (5.1, 12.2)	10.1 (4.3, 18.4)	15.0 (7.1, 25.8)^ab^	9.369	0.025
LP(a) (mg/L)	147.5 (113.3, 247.3)	160.0 (125.0, 253.0)	201.0 (135.5, 294.5)	265.0 (141.0, 363.5)^ab^	9.515	0.023
TG (mmol/L)	1.2 (0.8, 1.6)	1.2 (0.9, 1.9)	1.2 (0.9, 1.9)	1.5 (1.0, 2.1)	5.053	0.168
Glu (mmol/L)	7.17 ± 3.10	7.07 ± 2.93	6.82 ± 3.01	8.17 ± 3.54	1.676	0.172
HbA1c (%)	6.52 ± 1.67	6.31 ± 1.52	6.31 ± 1.46	6.52 ± 1.42	0.386	0.763
**IRA**						
LAD	29 (55.8)	63 (67.7)	46 (37.7)^b^	11 (33.3)^b^	23.433	< 0.001
LCX	16 (30.8)	6 (6.5)^a^	18 (14.8)	2 (6.1)^a^	18.332	< 0.001
RCA	3 (5.8)	19 (20.4)	58 (47.5)^ab^	19 (57.6)^ab^	44.767	< 0.001
**Lesion number**						
1	22 (42.3)	33 (35.5)	30 (24.6)	7 (21.2)	7.835	0.050
2	16 (30.8)	24 (21.1)	44 (36.1)	7 (21.2)	3.977	0.264
3	14 (26.9)	35 (37.6)	48 (39.3)	19 (57.6)^a^	8.065	0.045

a vs. Grade I, b vs. Grade II, c vs. Grade III, *P* < 0.05.

*W* women, *M* men, *HR* heart rate, *CRP* C-reactive protein, *Glu* blood glucose, *IRA* infarct-related artery, *RCA* right coronary artery, *LAD* left anterior descending coronary artery, *LCX* left circumflex coronary artery.

### Markers of myocardial injury and related parameters of cardiac function

#### Markers of myocardial injury

① The peak levels of hs-cTnT, CK-MB, and NT-proBNP in patients with grade IV were significantly higher than those in patients with grades I, II, and III. The peak levels of hs-cTnT, CK-MB, and NT-proBNP in patients with grade III were significantly higher than those in patients with grade I and II (*P* < 0.05). ② The peak levels of hs-cTnT and NT-proBNP in patients with grade II were significantly higher than those in patients with grade I (*P* < 0.05) (Table 2).

**Table 2:** Markers of myocardial injury [M (Q25, Q75)].

Grade	*n*	NT-proBNP (pg/ml)	hs-cTnT (ng/L)	CKMB (ng/ml)
I	52	892.1 (355.9, 1926.8)	1907.5 (739.1, 3752.3)	70.6 (23.5, 174.3)
II	93	1524 (860.4, 2979)^a^	3495 (1440.5, 6221.2)^a^	107.8 (39.8, 218.7)
III	122	2088 (1331, 3691.1)^ab^	5115.5 (2511.5, 7507)^ab^	151.5 (78.3, 285.9)^ab^
IV	33	3946 (1740.5, 10,559.5)^abc^	6315.7 (5175.5, 9624.5)^abc^	256.0 (150.0, 300.0)^abc^
*χ*^*2*^	–	51.706	49.079	32.649
*P*	–	< 0.001	< 0.001	< 0.001

a vs. Grade I, b vs. Grade II, c vs. Grade III, *P* < 0.05.

*NT-proBNP* N-terminal forebrain natriuretic peptide, *hs-cTnT* High-sensitivity troponin T, *CKMB* mb isotype of creatine kinase.

#### Cardiac function-related parameters

① The values of LVEDD and E/e′ in grade IV patients were significantly higher than those in grade I, II, and III patients. The values of LVEDD and E/e′ in grade III patients were significantly higher than those in grades I and II patients. The values of E/e′ in grade II patients were significantly higher than those in grade I patients (*P* < 0.05). ② The LVEF, LVFS, and E/A values in grade IV patients were significantly lower than those in grade I, II, and III patients. The values of LVEF, LVFS, and E/A in grade III patients were significantly lower than those in grades I and II. The values of LVEF and LVFS in grade II patients were significantly lower than those in grade I patients (*P* < 0.05) (Table 3).

**Table 3:** Cardiac function related parameters.

Functional parameters	I (*n* = 52)	II (*n* = 93)	III (*n* = 122)	IV (*n* = 33)	*F/χ*^*2*^	*P*
LVEDD	49.23 ± 3.39	49.78 ± 4.31	51.89 ± 4.99^ab^	54.21 ± 5.09^abc^	11.868	< 0.001
LVEF	55.5 ± 7.39	52.62 ± 6.84^a^	49.66 ± 6.49^ab^	44.48 ± 9.16^abc^	19.369	< 0.001
LVFS	29.9 ± 4.6	27.53 ± 4.02^a^	25.83 ± 4.04^ab^	22.21 ± 4.84^abc^	25.259	< 0.001
E/A	1.13 ± 0.48	1.08 ± 0.43	0.92 ± 0.28^ab^	0.77 ± 0.27^abc^	9.800	< 0.001
E/e′	11.62 ± 3.62	13.72 ± 4.94^a^	15.74 ± 5.91^ab^	18.98 ± 5.09^abc^	16.326	< 0.001

a vs. Grade I, b vs. Grade II, c vs. Grade III, *P* < 0.05.

*LVEDD* left ventricular end-diastolic dimension, *LVEF* left ventricular ejection fraction, *LVFS* left ventricular short-axis fractional shortening, *A* late diastolic, *E* early diastolic, *E/e′* early mitral inflow velocity to mitral early diastolic velocity ratio.

### Clinical outcomes

#### Major adverse cardiovascular events during one year

**Figure 1:**
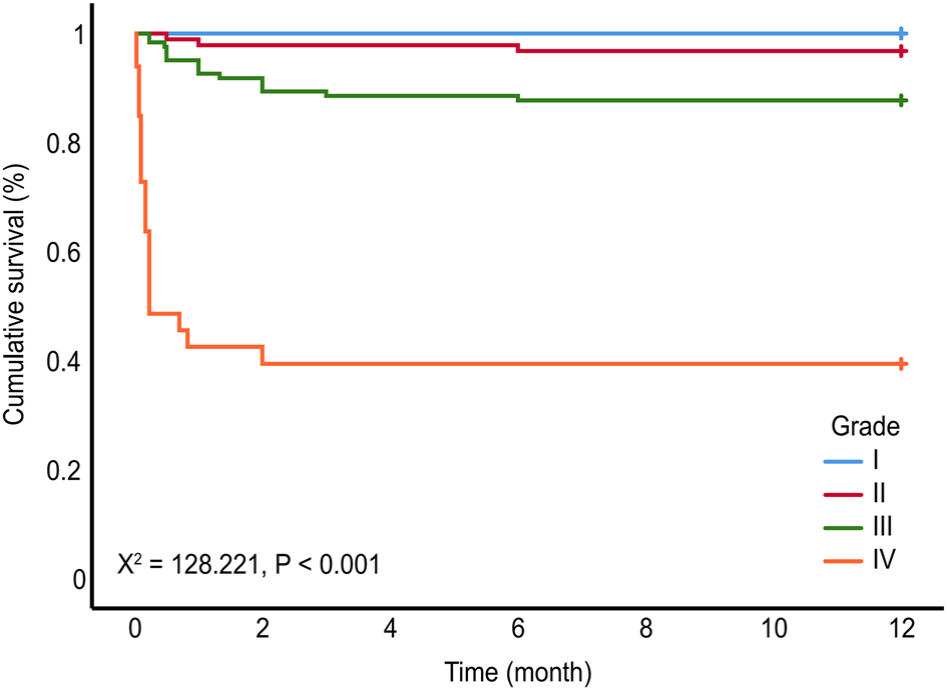
Kaplan‒Meier survival curves of patients with different grades.

**Figure 2:**
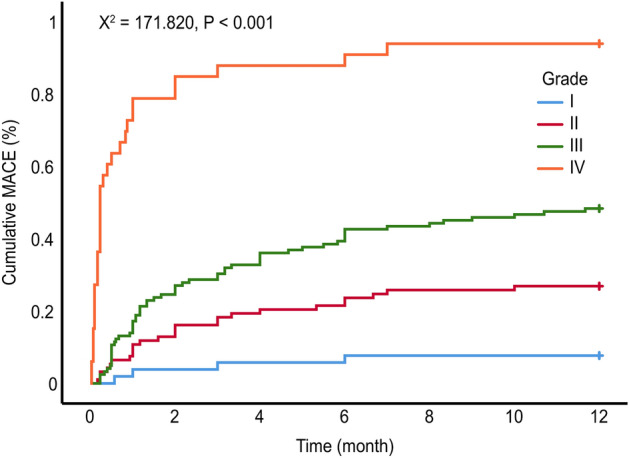
Kaplan‒Meier curves indicating composite MACE (angina, recurrent myocardial infarction, arrhythmia, heart failure, and all-cause death).

There was no significant difference in the proportion of patients with angina and recurrent myocardial infarction (MI) (*P* > 0.05) (Table 4). The frequencies of death, according to the grading, in the 1-year clinical follow-up were as follows: Grade I, 0%; II, 3.2%; III, 12.3%; and IV, 60.6% (*P*_*lograk*_ < 0.0001). The one-year cumulative MACE rate of patients at all grades was analyzed by the log-rank test: Total MACEs [I vs. II vs. III vs. IV: 7.7% (4/52) vs. 26.9% (25/93) vs. 48.4% (25/93) vs. 93.3% (31/33), *P*_*lograk*_ < 0.0001] (Figs. 1, 2).

**Table 4:** Incidence of MACEs in patients with different grades [*n* (%)].

Events	I (*n* = 52)	II (*n* = 93)	III (*n* = 122)	IV (*n* = 33)	*χ*^*2*^	*P*
Angina	3 (5.8)	14 (15.1)	20 (16.4)	1 (3.0)	7.017	0.071
Recurrent MI	0 (0.0)	1 (1.1)	2 (1.6)	0 (0.0)	0.992	1.000
Arrhythmia	0 (0.0)	2 (2.2)	11 (9.0)	4 (12.1)	10.471	0.009
Heart failure	1 (1.9)	4 (4.3)	11 (9.0)	6 (18.2)^a^	8.651	0.026
Death (all-cause)	0 (0.0)	3 (3.2)	15 (12.3)^a^	20 (60.6)^abc^	83.608	< 0.001
Total MACE	4 (7.7)	25 (26.9)^a^	59 (48.4)^ab^	31 (93.9)^abc^	73.035	< 0.001

a vs. Grade I, b vs. Grade II, c vs. Grade III, *P* < 0.05.

*MI* myocardial infarction, *MACE* major adverse cardiovascular events.

## Discussion

MIRI was first proposed by Jennings et al. in 1960^5^. Opening the IRA will inevitably lead to reperfusion injury. This study is the first to propose the MIRI manifestation grade to reflect the degree of MIRI. The study found that patients with RCA occlusions are more likely to have a higher degree of MIRI manifestation, followed by patients with LAD occlusions, and the LCX yields the least degree of MIRI. The reason is that the blood supply of the sinoatrial node and atrioventricular node mainly comes from the RCA, and RCA occlusion leads to inferior myocardial ischemia. There are abundant vagal nerve endings in the lower part of the heart. When the blood vessels are opened in PPCI, they cause the Bezold–Jarisch reflex^6^, which leads to sudden bradycardia and a sudden drop in blood pressure. After the recovery of IRA flow, the stress state is relieved, and vascular autonomic nerve regulation changes to the dominant position of the vagus nerve, leading to vasodilatation and then a decrease in blood pressure. Moreover, the occurrence of arrhythmia after reperfusion can also lead to a decrease in cardiac output. Previous clinical studies have shown that RCA and LCX occlusion can lead to acute inferior myocardial infarction. Because 80% of the patients are in the right coronary dominant type, when the IRA is the LCX (compared with the RCA), the infarct size is smaller, and the overall prognosis of patients is better^7^. The above conclusion is consistent with the conclusion that the myocardial ischemia‒reperfusion injury grade of patients with LCX occlusion is lower. Therefore, for patients with RCA culprit vessels, especially those with a higher MIRI grade, strategies to reduce reperfusion injury, such as systemic and intracoronary hypothermia and left ventricular unloading^8^, should be actively sought. In addition, remote ischemic conditioning (RIC)^9^, as the most promising myocardial protection strategy, can also be considered an auxiliary measure. Although RIC has not been promoted as a routine treatment, no studies have shown serious adverse effects in patients. For patients with a low MIRI grade, their medical compliance should be strengthened to achieve effective secondary prevention.

In this study, 38.7% of STEMI patients had multivessel disease, which was consistent with the 30–50% of STEMI patients with multivessel disease in previous studies^10^. For STEMI patients with multivessel disease, due to less collateral circulation and an insufficient blood supply, patients are prone to severe reperfusion injury, and the clinical manifestations of reperfusion injury are more obvious^11^. The mortality and MACE incidence of acute STEMI patients with multivessel disease are also significantly higher than those of patients with single-vessel disease^12^. In a later follow-up study, it was also found that patients with high MIRI manifestation grades have a high mortality and a high incidence of MACEs.

Inflammation plays an important role in the occurrence and development of atherosclerosis. Atherosclerosis itself is considered to be an inflammatory disease. CRP plays an important role in assessing the vulnerability of atherosclerotic plaques^13^. In this study, patients with higher MIRI grades also had higher CRP levels. The HDL-C level is an independent predictor of cardiovascular disease risk. Every 1 mg/dl decrease in HDL-C can increase the risk of coronary heart disease by 2–3%^14^. HDL-C levels were lower in patients with higher MIRI grades in the study. Lp(a) is a kind of lipoprotein containing cholesterol. In this study, the higher the MIRI grade, the higher was the LP(a) level. Patients with RCA, high CRP, high LP(a) and low HDL-C should be given close attention.

Byrne et al. measured the peak levels of CK-MB and cTnT in 1237 STEMI patients and evaluated the infarct size by single-photon emission computed tomography (SPECT) before discharge. The researchers found that the peak levels of CKMB and cTnT were significantly correlated with the infarct size and could predict mortality within 1 year^15^. The peak level of NT-proBNP was positively correlated with the severity of myocardial injury and the risk of adverse prognosis in patients with acute myocardial infarction^16^. In this study, by monitoring the peak levels of hs-cTnT, CKMB and NT-proBNP, we found that the higher the MIRI manifestation grade of STEMI patients, the higher was the biomarker level of myocardial injury and the lower was the grade of STEMI patients who also had myocardial injury.

After the occurrence of AMI, the regional wall motion of the infarcted area is abnormal, which manifests as local motion weakening, motion disappearance, and contradictory motion. From hours to days after AMI, due to the weakening or loss of myocardial contractile activity in the infarcted area, the local ventricular wall is thinned and extended, and the expansion of the heart cavity increases the left ventricular tension. The patients may exhibit early ventricular remodeling, which shows that the LVEDD has increased^17^. Ventricular remodeling is a common pathophysiological process after myocardial infarction and is related to the occurrence and development of heart failure^18^. Echocardiography is a reliable method for visual assessment of segmental and global cardiac function^19^. Using Doppler tissue imaging (TDI) to measure the ratio of the early diastolic blood flow peak (E) and the early diastolic movement speed (e′) of the mitral valve cusp at the level of the mitral valve cusp, it can be simple, effective and noninvasive to evaluate the left ventricular filling pressure of the heart and diastolic function^20,21^. This study showed that the higher the MIRI manifestation grade is, the larger the LVEDD and E/e′ and the smaller the E/A. The higher the MIRI manifestation grade is, the more obvious the early ventricular remodeling and diastolic dysfunction. LVEF and LVFS were measured by echocardiography to reflect ventricular systolic characteristics. In this study, the higher the MIRI manifestation grade, the lower were the LVEF and LVFS, indicating that the higher the MIRI manifestation grade, the more obvious was the left ventricular systolic dysfunction. In a recent study of young STEMI patients, lesion localization (LAD lesion, proximal lesion), no-reflow, and prolonged ischemic time appeared to be important determinants of LVEF decline^22^, which was slightly different from our results, considering that this may have been caused by population characteristics. Notably, no-reflow plays a significant role in predicting the prognosis of patients. Previous studies have shown that the atherogenic index of plasma (AIP) is independently associated with the no-reflow phenomenon in STEMI patients undergoing PPCI^23^. Future trials should combine these factors to evaluate STEMI outcomes as a whole.

At present, there are many methods for assessing the severity and prognosis of cardiovascular diseases. For example, a higher stress level may be accompanied by high mortality^24^, and imaging data can be used to more accurately predict the occurrence of MACEs^25^. Our MIRI grading system also showed good efficacy in the prognosis of STEMI patients. The higher the grade is, the higher the risk of death. The overall all-cause mortality rate of STEMI patients within one year after PCI was 12.67%, which was approximately the same as that in a previous study^26^ reporting that the mortality of STEMI patients within one year is approximately 12%. In addition, myocardial contractility in the IRA region of STEMI patients is decreased, and left ventricular remodeling leads to heart function damage. The higher the MIRI manifestation grade, the higher is the incidence of heart failure. The occurrence of heart failure is also an important factor that affects the prognosis of patients. This study found that there was no difference between the grades of patients with recurrent angina and recurrent myocardial infarction. The reason may be that the enhanced dual antiplatelet therapy (DAPT) after PCI in acute STEMI patients has a stronger inhibitory effect on platelet aggregation and effectively prevents recurrent myocardial infarction^27^. At the same time, the application of intensive lipid-lowering therapy^28^, β-receptor blockers, angiotensin-converting enzyme inhibitors (ACEIs), or angiotensin receptor antagonists (ARBs) also significantly reduced the incidence of angina and recurring myocardial infarction after STEMI^29^. We also consider that although MIRI is an important cause of death in STEMI patients, it also reflects the viable myocardium. Indeed, it does not affect the prognosis of cases, and we can refine the intervention of reperfusion. In this study, the differences in the incidence rate and cumulative survival rate of MACEs between grades I and II were not large, and consequently, the grade of MIRI manifestation can be further refined to allow for early intervention when a certain degree of MIRI manifestation is imminent or to avoid overtreatment when the degree is insufficient to affect the prognosis.

In summary, the MIRI grade can preliminarily be used to evaluate the short-term efficacy and long-term prognosis of PPCI in STEMI patients. During PPCI, prophylactic medication can reduce the degree of MIRI and further improve the efficacy and prognosis of PPCI. At the same time, patients with higher MIRI manifestation grades can be given more aggressive drug treatment. However, further research is needed to refine the grade.

## Limitations

First, the present investigation was a single-center study with a relatively small sample size and short follow-up time, which may have led to data bias. Second, there may have been measurement errors in the echocardiographic evaluation of cardiac function, and there are no follow-up echocardiographic data. A further evaluation of cardiac function and myocardial injury by MRI may be more convincing.

## Conclusions

The higher the MIRI manifestation grade is, the greater the degree of myocardial damage in patients and the higher the cumulative incidence of MACE within one year, especially in patients with multivessel disease, low HDL-C, high CRP, high LP(a) levels, and the RCA as the IRA.

## Data Availability

The raw data supporting the conclusions of this article will be made available by the authors, without undue reservation.
